# Academic self-perceptions and AI use among health professions students: the serial mediating roles of learned helplessness and academic-clinical learning loneliness

**DOI:** 10.3389/fpsyg.2026.1955625

**Published:** 2026-09-03

**Authors:** Pinar Ayyildiz

**Affiliations:** School of Foreign Languages, Yüksek Ihtisas University, Ankara, Türkiye

**Keywords:** academic and clinical learning loneliness, academic self-perceptions, academic wellbeing, artificial intelligence use, health professions students, impostor syndrome, learned helplessness

## Abstract

**Objective:**

This study inspected the direct and indirect relationships between academic self-perceptions and artificial intelligence (AI) use among health professions students. More specifically, it investigated if learned helplessness and academic and clinical learning loneliness independently and sequentially mediated the relationships of academic wellbeing, academic incompetence, impostor syndrome and overall academic self-perceptions with AI use.

**Methods:**

A quantitative, cross-sectional, and correlational design was employed. The sample consisted of 650 undergraduate health professions students aged 18–25 years. Data were collected employing the Academic Self-Perceptions Scale, Learned Helplessness Scale, Academic and Clinical Learning Loneliness Scale, and AI Use Scale. Descriptive statistics, Pearson correlations and serial mediation analyses were conducted using Hayes' PROCESS Model 6. A total of four diverse models were tested, with academic wellbeing, academic incompetence, impostor syndrome and overall academic self-perceptions entered as predictors. Indirect effects were estimated using 5,000 bootstrap samples and 95% bias-corrected confidence intervals.

**Results:**

Academic wellbeing and positive overall academic self-perceptions were negatively associated with learned helplessness, academic and clinical learning loneliness, and AI use. In contrast, academic incompetence and impostor syndrome were positively linked to all three variables. Learned helplessness positively predicted academic and clinical learning loneliness, and both mediators were positively ascribed to AI use. All specific indirect effects through learned helplessness and academic and clinical learning loneliness were significant. The serial indirect effects through learned helplessness followed by academic and clinical learning loneliness were also significant in all four models, indicating partial mediation.

**Discussion:**

The findings spotlight that AI use of health professions students is related not only to technological accessibility but also to students' academic self-evaluations, perceived control over learning, and connection with academic and clinical learning environments. Positive academic self-perceptions can reduce AI use by limiting helplessness and learning-related loneliness, whereas academic incompetence and impostor syndrome may increase AI use through the opposite process. The results highlight the significance of supporting academic competence, learner agency, relational connection, and responsible AI use in health professions education.

## 1 Introduction

The rapid diffusion of generative artificial intelligence (GenAI) has begun to transform academic spheres along with the production and dissemination of scientia all around the world. How university students access information, solve problems, complete assignments and regulate their learning are of no difference; they are affected in this manner as well. This “reform-like” period is particularly consequential in health professions education, for which students must integrate extensive theoretical knowledge with clinical reasoning, practical skills, ethical responsibility, and patient-centered decision-making. Recent evidence indicates that health professions students use GenAI for concept clarification, information retrieval, writing support, practice activities, and the simulation of clinical scenarios (Pham et al., 2025). Nursing students similarly describe GenAI as an accessible and efficient learning resource that can simplify complex content and provide immediate academic assistance (Han et al., 2025). Nevertheless, the increasing availability of AI-supported learning also raises concerns about inaccurate information, academic integrity, cognitive outsourcing, reduced critical evaluation, and excessive technological reliance (Preiksaitis and Rose, 2023; Zhai et al., 2024). The implications of AI use cannot be understood solely in terms of technological acceptance or digital competence. Students do not engage with AI in a psychological vacuum; rather, their patterns of use may reflect how they perceive themselves, interpret academic challenges, and experience their learning environment. Namely, AI-supported learning does not occur *in vacuo*, it is embedded within learner perceptions of competence, control and relational support. The line of research in higher education has traditionally dwelled on technology adoption through perceived usefulness, ease of use, behavioral intention, or AI literacy. Admitting these factors remain essential, they present solely a partial account of why some students use AI as a complementary learning tool whereas others may increasingly rely on it when they feel academically inadequate, unsupported, or unable to cope independently. Evidence that GenAI use can simultaneously improve efficiency and intensify technological dependence illustrates this tension (Zhang and Xu, 2024). Among nursing students, AI has been associated with educational benefits together with reduced critical thinking, dependency-related concerns, and emotional discomfort when access is unavailable (Saglam and Kalanlar, 2025). Accordingly, the psychological conditions underlying AI use require closer examination. This issue is particularly relevant to health professions students because their education involves overlapping academic and clinical demands. Students must cope with intensive coursework, frequent assessment, clinical placements, interpersonal evaluation, and the possibility that mistakes may affect patient care. Medical and dental students commonly report substantial academic stress, and the perceived quality of their educational environment is associated with both their quality of life and academic performance (Wahid et al., 2023). Nursing students experience additional clinical stressors involving fear of making errors, harming patients, interacting with healthcare staff, and performing under observation (Mohamed et al., 2024). The clinical learning environment and the conduct of instructors and healthcare personnel can therefore influence students' stress, engagement, and willingness to participate in learning activities (Mazalová et al., 2022). Within these demanding environments, students' interpretations of their own academic functioning become especially critical. Academic self-perceptions influence whether challenges are interpreted as manageable developmental experiences or as evidence of enduring personal inadequacy. In health professions education, academic self-perceptions have been associated with behavioral and cognitive engagement, which in turn, can facilitate academic achievement (Kassab et al., 2024). Positive psychological resources such as wellbeing, resilience, and self-efficacy support students' capacity to adapt to academic and clinical demands (Chow et al., 2018; Aryuwat et al., 2023). Conversely, negative self-appraisals, doubts about competence, and difficulty internalizing success may heighten vulnerability to stress, withdrawal, and maladaptive coping.

One prominent manifestation of negative academic self-appraisal is the impostor phenomenon. Impostor feelings are widespread among medical, nursing, and dental students and are associated with stress, anxiety, depression, low self-esteem, diminished resilience, and poor sense of belonging (Naser et al., 2022; El-Ashry et al., 2024; Jansson et al., 2025). A recent scoping review reported that the prevalence of impostor experiences among medical undergraduates ranged from approximately 31% to 76%, depending on the sample and measurement approach (Chua et al., 2025). Such findings suggest that academic success does not necessarily protect students from perceived incompetence. Students may meet formal performance requirements while continuing to believe that they lack the ability needed for future academic or clinical tasks. When perceptions of inadequacy are repeatedly reinforced, students may begin to question whether their effort can meaningfully influence academic outcomes. This pattern is consistent with learned helplessness, a state characterized by diminished perceived control, reduced persistence, and an expectation that personal action will not change adverse circumstances. Learned helplessness has been observed among nursing students and has been associated with low programme satisfaction, involuntary programme choice, financial disadvantage, and poor interpersonal relationships with classmates (Wang et al., 2025). Feelings of helplessness during clinical education are also linked with psychological strain and difficulty coping with challenging placements (Dresen et al., 2025). Thus, learned helplessness may provide an explanatory bridge between students' academic self-perceptions and their subsequent withdrawal from interpersonal and learning-related resources.

Such withdrawal might foster loneliness within academic and clinical learning environments. Despite the fact that loneliness is often treated as a general social or emotional condition, health professions students may experience a more context-specific form of disconnection. They may feel unable to discuss academic difficulties, reluctant to ask questions in clinical settings, excluded from peer learning, or unsupported by instructors and clinical supervisors. Social isolation among health science students has been associated with competitive programme climates, weak support systems, excessive coursework, strained faculty relationships, and limited opportunities to communicate with peers (Ray et al., 2019). Loneliness is also highly prevalent among nursing students and appears to constitute a distinct psychological construct rather than merely a derivative of anxiety or depression (Mi et al., 2025). Furthermore, loneliness is negatively associated with self-directed learning, suggesting that relational disconnection may undermine students' capacity to initiate and sustain learning independently (Zhao, 2022). These conditions may create a pathway through which students increasingly turn to AI as an immediately available and non-judgemental source of assistance. AI tools do not display impatience, directly evaluate students in front of peers, or require students to disclose perceived incompetence to an instructor. For students who feel helpless or isolated, these characteristics may make AI particularly attractive. On the other hand, repeated substitution of AI for human academic interaction may also reinforce individualized learning patterns and reduce opportunities for discussion, peer collaboration, and help-seeking. A systematic review of GenAI use in health professions education found that GenAI was commonly used for individual activities such as inquiry, practice, production, and knowledge acquisition, whereas discussion and collaboration were substantially less represented (Pham et al., 2025). Reviews of AI and student wellbeing similarly warn that unbalanced use may result in reduced face-to-face interaction, social isolation, and weakened interpersonal engagement (Klimova and Pikhart, 2025). Despite these emerging findings, academic self-perceptions, learned helplessness, learning-related loneliness, and AI use have largely developed as separate areas of research. Studies on impostor phenomenon generally focus on prevalence and psychological distress; research on learned helplessness often examines burnout, failure, or mental health; and studies of loneliness typically use broad measures of social or emotional isolation. Meanwhile, AI research predominantly concentrates on adoption, usefulness, attitudes, literacy, or dependency. Limited attention has been paid to the sequential psychological process through which students' perceptions of their academic selves may influence their use of AI.

This research plans to addresses this gap by studying whether learned helplessness and academic and clinical learning loneliness sequentially mediate the relationship between academic self-perceptions and AI use among health professions students. Academic self-perceptions are represented through academic wellbeing, impostor phenomenon and academic incompetence. An Academic and Clinical Learning Loneliness Scale was also developed to capture disconnection arising specifically in academic collaboration, classroom learning, clinical practice, supervision, and help-seeking. By integrating academic self-evaluation, perceived control, relational learning experiences, and technology-related behavior, the study seeks to offer a psychologically grounded explanation of AI use in health professions education.

### 1.1 Theoretical framework and literature review

#### 1.1.1 Academic self-perceptions in health professions education

Academic self-perceptions refer to students' evaluations of their own functioning, competence, adjustment, and psychological experience within the educational environment. Such perceptions affect students' expectations, emotional reactions, learning strategies, persistence, and engagement. Social cognitive perspectives propose that students' beliefs about their capabilities shape the goals they establish, the effort they invest, and their persistence when they encounter difficulty. In the present study, Social Cognitive Theory provides an overarching theoretical framework for organizing the proposed relationships. Social Cognitive Theory emphasizes the reciprocal interplay among personal cognitions, behavioral patterns, and environmental conditions and assigns a central role to efficacy-related beliefs in regulating motivation, persistence, and action (Bandura, 1986, 1997). Within this perspective, academic self-perceptions represent students' self-referential appraisals of their capacity to manage academic and clinical demands. Persistently negative appraisals may be associated with diminished perceived control and weaker expectations that personal effort will produce desired outcomes, thereby creating conditions compatible with learned helplessness. Reduced perceived control may, in turn, be linked with lower engagement in help-seeking and interpersonal learning resources, while AI use can be positioned as a behavioral correlate of these interconnected academic, motivational, and relational experiences. Research with medical students has shown that motivation influences learning engagement and academic performance, while efficacy-related beliefs form part of this motivational process (Wu et al., 2020). Among broader university populations, adaptive coping profiles are similarly associated with stronger efficacy beliefs, indicating that students' self-perceptions are embedded in how they respond to academic stressors (Freire et al., 2020). Health professions education may intensify the importance of academic self-perceptions as students are repeatedly exposed to evaluation in both academic and clinical domains. They must demonstrate theoretical knowledge, procedural competence, communication skills, and clinical judgement, often while being observed by instructors, peers, patients, and healthcare professionals. Positive perceptions of the educational environment promote engagement and achievement, whereas adverse learning experiences may undermine students' academic functioning (Kassab et al., 2024). Clinical practice adds further pressures, including uncertainty, fear of mistakes, concerns about patient safety, and demanding interactions with supervisors (Mohamed et al., 2024). Consequently, health professions students may hold positive perceptions in one area of academic life while simultaneously experiencing substantial doubts in another. In the present study, academic self-perceptions are conceptualized through three dimensions: academic wellbeing, impostor phenomenon, and academic incompetence. These dimensions represent related but varying aspects of students' academic experience. Academic wellbeing reflects positive adjustment and functioning. Impostor phenomenon captures difficulty internalizing genuine achievement. Academic incompetence reflects a more direct perception of being unable to satisfy academic expectations. Considering these dimensions together makes it possible to examine academic self-perceptions without reducing them to either positive wellbeing or negative self-doubt alone.

#### 1.1.2 Academic wellbeing

Academic wellbeing can be understood as positive psychological functioning within educational life, encompassing satisfaction, emotional balance, meaningful engagement, manageable academic demands, and a sense of effective participation. It is not simply the absence of distress. Students may report low clinical anxiety while still feeling disengaged, unsupported, or dissatisfied; conversely, they may experience temporary stress while maintaining a strong sense of purpose and academic functioning. This distinction corresponds with approaches that treat mental distress and positive wellbeing as related but non-identical dimensions. Health professions students' wellbeing is affected by both individual and environmental resources. Resilience is positively associated with perceived wellbeing among nursing students (Chow et al., 2018), while academic stress is negatively related to subjective wellbeing (Ma, 2023). Research across nursing programmes also indicates that wellbeing supports academic success and may reduce burnout and attrition (Uzzell et al., 2025). Conversely, psychological distress, poor sleep, and elevated academic stress can impair students' psychosocial functioning and academic experiences (Alhamed, 2023). Academic wellbeing also appears to be closely linked with how students perceive their competence and available support. Self-compassion is associated with higher academic self-efficacy and lower academic stress among nursing students (Nazari et al., 2025). Supportive educational environments can foster resilience through mentoring, interpersonal resources, practical preparation, and positive role modeling (Chye et al., 2024). Therefore, academic wellbeing may help students maintain perceived control in the face of difficulty, reducing the likelihood that setbacks will be generalized into stable expectations of helplessness.

#### 1.1.3 Impostor phenomenon

The impostor phenomenon describes persistent self-doubt and difficulty internalizing achievement despite objective evidence of competence. Individuals experiencing impostor feelings may believe that their success is attributable to luck, exceptional effort, favorable circumstances, or others' mistaken evaluations. They may also fear that future performance will reveal their supposed inadequacy. The phenomenon is therefore not synonymous with actual low achievement; it concerns a discrepancy between external indicators of competence and internal perceptions of legitimacy. Impostor phenomenon is widely documented across health professions education. Among Swedish medical students, approximately 58% met the study threshold for impostor phenomenon, and impostor scores were inversely associated with resilience (Kristoffersson et al., 2024; Yilmaz and Serin, 2026). In a multidisciplinary sample including medical, dental, and nursing students, 64% exceeded the specified threshold, with impostor scores positively associated with stress and anxiety (Jansson et al., 2025). A multicentre study of nursing students also found substantial moderate, frequent, and intense impostor experiences and reported a strong association with depression, anxiety, and stress (El-Ashry et al., 2024). Studies in medical education further connect impostor phenomenon with diminished self-compassion, sociability, self-esteem, and peer adjustment (Rosenthal et al., 2021). Low self-esteem has been identified as a strong predictor of impostor phenomenon among medical students studying in an international educational environment (Naser et al., 2022). Research among clinical medical students also associates impostor experiences with stress, depression, and anxiety during the transition into clinical education (Shinawatra et al., 2023). These findings are reinforced by review evidence showing that impostor phenomenon in medical education is associated with transition periods, perceived poor clinical skills, inadequate faculty support, burnout, and weak belonging (Chua et al., 2025). The association between impostor phenomenon and social belonging is particularly relevant to the present study. Students who fear being exposed as incompetent may avoid asking questions, disclosing uncertainty, or seeking assistance since these behaviors could be interpreted as confirmation of inadequacy. This avoidance may reduce access to corrective feedback and supportive interaction. Over time, the student may become more isolated while continuing to interpret the absence of mastery as evidence of incompetence. Thus, impostor phenomenon has the potential to effectuate learned helplessness both through negative competence appraisals and through the avoidance of interpersonal resources that could challenge those appraisals.

#### 1.1.4 Academic incompetence

Academic incompetence refers to students' subjective judgement that they do not possess the knowledge, skills, or capacity called for to satisfy educational expectations. It differs from objective academic performance for students might perceive themselves as incompetent even when their grades are satisfactory. It also differs from impostor phenomenon: impostor phenomenon centers on the inability to accept demonstrated success, whereas academic incompetence reflects a more direct belief that one's academic capacity is inadequate. Perceived competence is central to adaptive motivation and learning persistence. Students who perceive themselves as capable are more likely to engage, seek feedback, and persist in difficult tasks. By contrast, low efficacy-related perceptions are associated with academic burnout, reduced initiative, and psychological distress among nursing students (Chen et al., 2023; Zhu et al., 2023). Academic self-efficacy is negatively related to academic stress and burnout, while self-compassion and adaptive support may strengthen competence beliefs (Nazari et al., 2025). Loneliness has also been found to negatively predict academic self-efficacy, demonstrating that competence perceptions and relational experiences may influence one another (Aslan and Polat, 2024). In clinical education, perceived incompetence may become especially salient on account of the fact that students must perform under supervision and translate theoretical knowledge into action. Clinical uncertainty, fear of error, and concern about negative evaluation may lead students to interpret normal learning difficulties as proof that they are unsuitable for the profession. When these interpretations persist across academic and clinical settings, students may increasingly expect poor outcomes regardless of effort. Academic incompetence may therefore represent a necessary cognitive antecedent of learned helplessness.

#### 1.1.5 Academic self-perceptions and learned helplessness

Learned helplessness emerged from the observation that repeated exposure to uncontrollable adverse outcomes can produce motivational, cognitive, and emotional deficits. In academic settings, helpless students may believe that achievement is largely beyond their control. They may consequently reduce effort, avoid difficult tasks, disengage from feedback, or fail to use available support. The central issue is not simply that a task is difficult; it is the expectation that personal action will not alter the outcome. Academic self-perceptions are theoretically relevant to this expectation. Positive academic wellbeing may preserve perceived control by helping students view setbacks as temporary and manageable. Stronger competence perceptions similarly support persistence and adaptive strategy use. In contrast, impostor feelings and perceptions of academic incompetence may encourage stable, internal, and generalized explanations for difficulty. When students repeatedly interpret setbacks as evidence that they fundamentally lack ability, expectations of uncontrollability may become more likely. Empirical findings support elements of this proposed relationship. Nursing students with stronger self-efficacy report lower learning burnout, whereas anxiety and depression may bring about burnout partly through weakened efficacy beliefs (Zhu et al., 2023). Fear of failure and maladaptive failure appraisal have also been linked to learned helplessness among novice nursing students (Taha et al., 2025). Among vocational nursing students, low programme satisfaction and non-voluntary programme choice are associated with higher learned helplessness, suggesting that diminished agency and negative academic evaluation are relevant antecedents (Wang et al., 2025). Moreover, self-compassion may buffer the association between learned helplessness and adverse mental health outcomes among university students (Xue et al., 2023). Based on this theoretical and empirical foundation, positive academic self-perceptions are expected to reduce learned helplessness, whereas impostor phenomenon and academic incompetence are expected to increase it.

**H1a:** Academic wellbeing is negatively associated with learned helplessness.

**H1b:** Impostor phenomenon is positively associated with learned helplessness.

**H1c:** Academic incompetence is positively associated with learned helplessness.

**H1d:** Overall academic self-perceptions are negatively associated with learned helplessness.

#### 1.1.6 Academic self-perceptions and academic and clinical learning loneliness

Academic and clinical learning loneliness refers to a perceived lack of meaningful connection, support, accessibility, and belonging within academic and clinical learning processes. It includes feeling alone when attempting to understand course content, perceiving that one cannot discuss academic difficulties with peers or instructors, lacking support during clinical practice, and hesitating to seek guidance due to anticipated judgement. This construct is narrower than general loneliness. A student may have an active social life yet feel isolated when completing academic tasks or entering clinical settings. Conversely, a student may have few social relationships at any rate feel adequately supported in learning. Existing loneliness instruments generally focus on emotional or social connection and may not fully represent relational deficits tied to teaching, supervision, collaborative learning, and clinical practice. Evidence from health professions education demonstrates that such context-specific isolation is plausible. Health science students have identified exclusionary programme climates, competition, limited faculty relationships, excessive workload, and weak support as sources of social isolation (Ray et al., 2019). Nursing students' loneliness is negatively associated with self-directed learning (Zhao, 2022), while perceived sociability in online learning and social intelligence are negatively associated with loneliness (Savci et al., 2022). Among health science students, loneliness has also been linked to academic factors and relationships with peers and family (Mangali et al., 2025). Academic self-perceptions may influence these experiences through help-seeking and belonging. Students with stronger wellbeing and competence perceptions may be more willing to participate, disclose uncertainty, and use academic support. Conversely, students who perceive themselves as impostors or academically incompetent may fear that seeking help will expose their inadequacy. Rosenthal et al. (2021) found that impostor phenomenon was associated with lower sociability, reduced peer adjustment, and greater loneliness-related characteristics. Review evidence also connects impostor phenomenon with interpersonal conflict and a weak sense of belonging in medical education (Chua et al., 2025). Thus, academic self-perceptions may directly shape students' experience of academic and clinical learning loneliness.

**H2a:** Academic wellbeing is negatively associated with academic and clinical learning loneliness.

**H2b:** Impostor phenomenon is positively associated with academic and clinical learning loneliness.

**H2c:** Academic incompetence is positively associated with academic and clinical learning loneliness.

**H2d:** Overall academic self-perceptions are negatively associated with academic and clinical learning loneliness.

#### 1.1.7 Learned helplessness and academic and clinical learning loneliness

Learned helplessness can give rise to learning loneliness by reducing students' willingness to initiate interaction and seek support. Students who believe that their actions will not improve outcomes may conclude that asking questions, attending support sessions, or discussing difficulties is unlikely to help. This behavioral withdrawal can reduce opportunities for feedback, peer collaboration, and corrective learning experiences. The social environment also shapes helplessness. Poor interpersonal relationships with classmates have been associated with learned helplessness among nursing students (Wang et al., 2025), while school belonging and exclusion influence the development of helplessness in educational settings (Raufelder and Kulakow, 2022). These findings suggest that helplessness and relational disconnection may operate recursively: inadequate support may intensify helplessness, and helplessness may further reduce engagement with available support. The ordering specified in the present model should therefore be understood as a theoretically hypothesized analytical sequence rather than as an empirically established temporal ordering. Learned helplessness is positioned before learning loneliness owing to the fact that diminished perceived control may plausibly be associated with reduced help-seeking and engagement with available academic support, while reverse and reciprocal relationships remain theoretically possible. Within clinical education, this process may be particularly consequential. Students who feel unable to control outcomes may hesitate to request supervision, admit uncertainty, or participate actively in clinical teams. Such withdrawal may then strengthen their perception that they are learning alone. Accordingly, learned helplessness is expected to predict greater academic and clinical learning loneliness.

**H3:** Learned helplessness is positively associated with academic and clinical learning loneliness.

#### 1.1.8 Academic and clinical learning loneliness and AI use

GenAI provides students with immediate, personalized, and relatively low-risk access to academic assistance. It can explain concepts repeatedly, generate examples, assist with writing, and respond without visible social judgement. These features are educationally valuable, but they may be particularly attractive to students who feel disconnected from peers, instructors, or clinical supervisors. Evidence from health professions education demonstrates that GenAI is frequently used for individualized learning activities. Practice, inquiry, production, and knowledge acquisition are the most represented uses, whereas discussion and collaboration receive considerably less attention (Pham et al., 2025). Health sciences students value GenAI for customized learning, efficiency, language assistance, and autonomous study, but they also express concern about over-reliance and reduced interpersonal engagement (Fawaz et al., 2025). Nursing students report similar benefits alongside concerns regarding accuracy, critical thinking, and dependence (Han et al., 2025; Saglam and Kalanlar, 2025). Learning loneliness may therefore increase AI use through a compensatory mechanism. When human support feels inaccessible, insufficient, or threatening, AI can function as an alternative academic interlocutor. This does not imply that AI use is inherently problematic. Rather, its function may differ across students: some use it to extend active learning, while others may use it to replace help-seeking, collaboration, or independent problem-solving. Research on related digital behaviors supports this possibility. Loneliness mediates associations involving internet addiction, academic burnout, smartphone addiction, and digital engagement (Alinejad et al., 2022; Gu et al., 2023). In spite of the fact that the results cannot be transferred directly to GenAI *per se*, they still suggest that relational needs may influence the use of readily accessible technologies. Emerging research also indicates a complex relationship between AI use, loneliness, academic stress, and efficacy beliefs (Wang et al., 2025). Consequently, academic and clinical learning loneliness is expected to be associated with greater AI use, particularly when AI serves a compensatory or dependency-related function.

**H4:** Academic and clinical learning loneliness is positively associated with AI use.

#### 1.1.9 Learned helplessness and AI use

Learned helplessness may also influence AI use independently of loneliness. Students who doubt their ability to solve problems may delegate difficult cognitive tasks to AI. Given that GenAI can rapidly generate answers, explanations, and completed textual outputs, it may reduce the immediate discomfort associated with uncertainty or perceived incompetence. Repeated reliance on this strategy may reinforce the belief that academic tasks cannot be completed without technological assistance. Systematic evidence suggests that over-reliance on AI dialogue systems can encourage uncritical acceptance of AI recommendations and weaken analytical reasoning, decision-making, and critical thinking (Zhai et al., 2024). Generative AI use may simultaneously improve efficiency and self-confidence while increasing technological dependence (Zhang and Xu, 2024). Among nursing students, AI-related dependency and distress in the absence of AI have already been documented (Saglam and Kalanlar, 2025). Academic stress has also been associated with GenAI dependency through cognitive and emotional mechanisms such as performance expectations and academic anxiety (Liu et al., 2026). These findings suggest that learned helplessness may be associated with more frequent academic AI use, while cognitive outsourcing and dependency represent possible mechanisms that cannot be directly inferred from the present AI-use measure. Students who perceive their own actions as ineffective may therefore be more inclined to use AI-supported assistance while the specific motivation underlying such use cannot be determined from frequency-based scores alone.

**H5:** Learned helplessness is positively associated with AI use.

#### 1.1.10 Academic self-perceptions and AI use

Academic self-perceptions may influence AI use both directly and through learned helplessness and learning loneliness. Students with stronger academic wellbeing may use AI strategically while retaining independent judgement and human learning relationships. Students experiencing impostor phenomenon or academic incompetence may instead use AI to avoid exposing uncertainty, obtain reassurance, compensate for perceived deficits, or complete tasks they believe they cannot manage independently. Current research indicates that AI use does not have a uniformly positive or negative psychological meaning. GenAI can promote confidence and efficiency, yet frequent use may also increase dependence (Zhang and Xu, 2024). AI-supported learning may offer personalization and accessibility while generating technostress, social isolation, and reduced interpersonal interaction when integration is unbalanced (Klimova and Pikhart, 2025). Accordingly, the relationship between academic self-perceptions and AI use should be interpreted in relation to the function measured by the AI scale. Bearing in mind higher scores on the AI Use Scale indicate more frequent or intensive academic AI use rather than dependency or compensatory motivation, the proposed associations should be interpreted in terms of differences in the frequency of academic AI use.

**H6a:** Academic wellbeing is negatively associated with AI use.

**H6b:** Impostor phenomenon is positively associated with AI use.

**H6c:** Academic incompetence is positively associated with AI use.

**H6d:** Overall academic self-perceptions are negatively associated with AI use.

#### 1.1.11 The mediating role of learned helplessness

Academic self-perceptions may shape AI use indirectly by influencing students' perceived control over learning. Students with positive academic wellbeing are more likely to interpret difficulty as manageable. Conversely, impostor feelings and academic incompetence may increase the expectation that personal effort will not produce successful outcomes. This expectation may encourage students to transfer academic tasks to AI or seek externally generated solutions. Learned helplessness has previously been examined as a mediator between efficacy-related beliefs and academic burnout (Yang and Tu, 2024). Related evidence shows that helplessness is linked with mental strain during clinical practice (Dresen et al., 2025) and that maladaptive failure appraisals induce helplessness among nursing students (Taha et al., 2025). These findings provide a basis for expecting helplessness to transmit the effects of academic self-perceptions to AI use.

**H7a:** Learned helplessness mediates the relationship between academic wellbeing and AI use.

**H7b:** Learned helplessness mediates the relationship between impostor phenomenon and AI use.

**H7c:** Learned helplessness mediates the relationship between academic incompetence and AI use.

**H7d:** Learned helplessness mediates the relationship between overall academic self-perceptions and AI use.

#### 1.1.12 The mediating role of academic and clinical learning loneliness

Academic self-perceptions may also influence AI use by shaping students' connection with their learning environment. Students who feel academically secure may engage more readily with peers, instructors, and supervisors. Those who experience impostor phenomenon or perceived incompetence may avoid interpersonal learning depending of fear of judgement. AI may then become an alternative source of guidance. This proposition is supported by evidence linking impostor phenomenon with weak belonging and interpersonal difficulties (Chua et al., 2025), loneliness with reduced self-directed learning (Zhao, 2022), and loneliness with technology-related dependency behaviors (Alinejad et al., 2022; Gu et al., 2023). Academic and clinical learning loneliness may therefore explain why negative academic self-perceptions are associated with greater AI use.

**H8a:** Academic and clinical learning loneliness mediates the relationship between academic wellbeing and AI use.

**H8b:** Academic and clinical learning loneliness mediates the relationship between impostor phenomenon and AI use.

**H8c:** Academic and clinical learning loneliness mediates the relationship between academic incompetence and AI use.

**H8d:** Academic and clinical learning loneliness mediates the relationship between overall academic self-perceptions and AI use.

#### 1.1.13 Serial mediation of learned helplessness and academic and clinical learning loneliness

The proposed serial model specifies learned helplessness before academic and clinical learning loneliness as a theoretically hypothesized analytical ordering rather than as an established temporal sequence. From a social cognitive perspective, negative academic self-perceptions may be associated with diminished perceived control, which may reduce help-seeking and engagement with available learning resources (Bandura, 1986, 1997). Reduced engagement may, in turn, be associated with greater academic and clinical learning loneliness and more frequent reliance on AI-supported assistance. Howbeit, reverse and reciprocal relationships remain theoretically possible, and the present cross-sectional design cannot establish temporal precedence between learned helplessness and learning loneliness. Accordingly, the serial pathway tested here represents one theoretically specified configuration of these interrelated processes.

**H9a:** Learned helplessness and academic and clinical learning loneliness serially mediate the relationship between academic wellbeing and AI use.

**H9b:** Learned helplessness and academic and clinical learning loneliness serially mediate the relationship between impostor phenomenon and AI use.

**H9c:** Learned helplessness and academic and clinical learning loneliness serially mediate the relationship between academic incompetence and AI use.

**H9d:** Learned helplessness and academic and clinical learning loneliness serially mediate the relationship between overall academic self-perceptions and AI use.

### 1.2 Purpose of the study and research model

**Figure 1:**
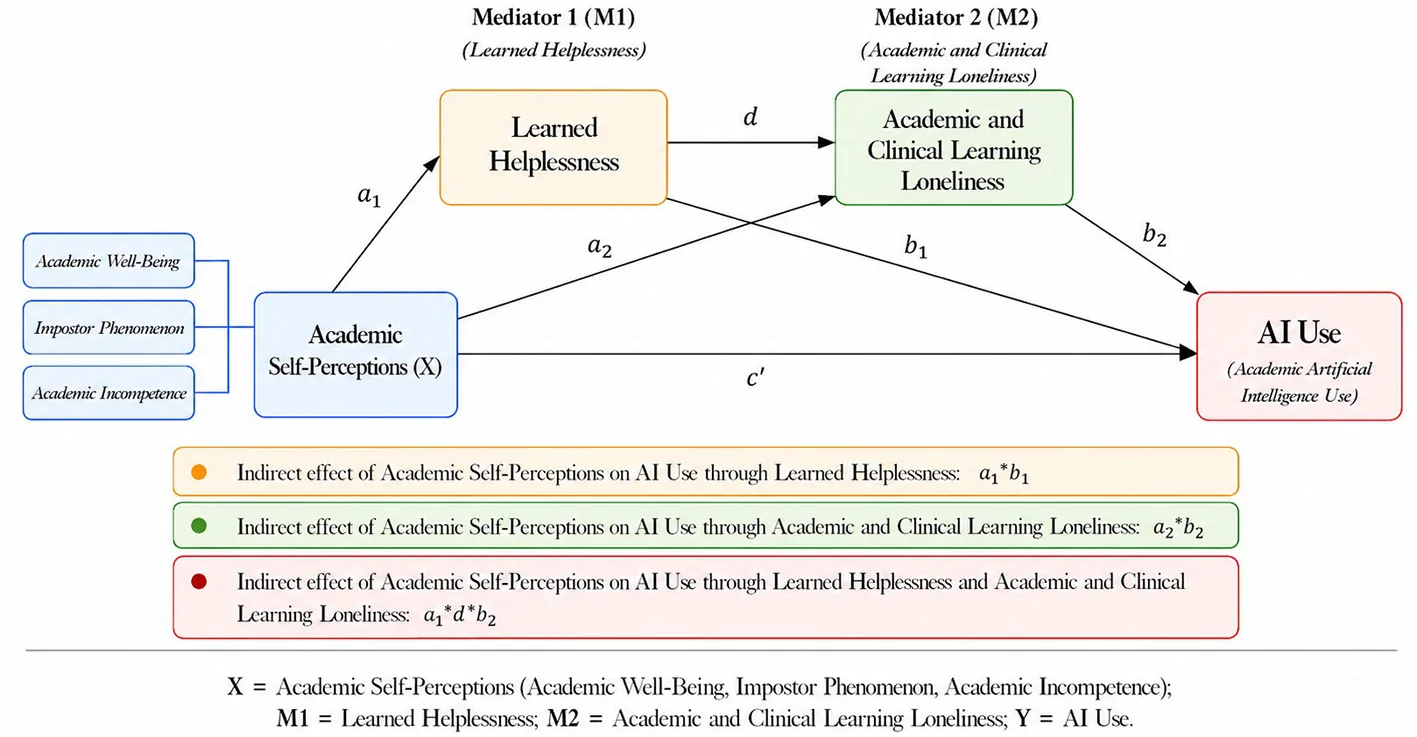
Research model.

Academic self-perceptions refer to students' evaluations of their own functioning, competence, adjustment, and psychological experience within the educational environment. The present study aims to examine the direct and indirect relationships between academic self-perceptions and AI use among health professions students. Academic self-perceptions are represented by three dimensions: academic wellbeing, impostor phenomenon, and academic incompetence. The study investigates the independent and serial mediating roles of learned helplessness and academic and clinical learning loneliness in these relationships. The proposed model is based on the assumption that students' academic self-perceptions may first influence their levels of learned helplessness and subsequently shape their experiences of academic and clinical learning loneliness. In this process, negative academic self-perceptions may weaken students' perceived control over academic outcomes, which may, in turn, increase their withdrawal from academic and clinical learning relationships. Such relational disconnection may strengthen students' tendency to use AI as an alternative source of academic support. Accordingly, the research model examines the direct, indirect, and serial relationships among academic self-perceptions, learned helplessness, academic and clinical learning loneliness, and AI use within an integrated framework. The proposed research model is presented in Figure 1.

Figure 1 presents the proposed serial mediation model linking academic self-perceptions to AI use. Academic self-perceptions, represented by academic wellbeing, impostor phenomenon, and academic incompetence, are hypothesized to influence AI use both directly and indirectly. The model includes learned helplessness as the first mediator and academic and clinical learning loneliness as the second mediator. Accordingly, three indirect pathways are examined: through learned helplessness, through academic and clinical learning loneliness, and sequentially through learned helplessness followed by academic and clinical learning loneliness.

## 2 Materials and methods

### 2.1 Research design

This study employed a quantitative, cross-sectional, and correlational research design to examine the relationships among academic self-perceptions, learned helplessness, academic and clinical learning loneliness, and AI use among health professions students. Cross-sectional designs allow researchers to examine associations among variables measured within a defined period albeit the resulting relationships should not be interpreted as definitive evidence of temporal or causal effects (Spector, 2019). The proposed model was tested within a serial mediation framework. Academic self-perceptions, represented by academic wellbeing, impostor phenomenon, and academic incompetence, were specified as the predictor variables; learned helplessness and academic and clinical learning loneliness were included as the first and second mediators, respectively; and AI use was specified as the outcome variable. Serial mediation analysis enables the examination of an indirect process in which a predictor is associated with an outcome through two or more mediators operating in a theoretically specified sequence (Hayes, 2022). Accordingly, the design allowed the examination of direct effects, specific indirect effects through each mediator, and serial indirect effects operating through learned helplessness and academic and clinical learning loneliness.

### 2.2 Participants

The study sample consisted of 650 undergraduate health professions students aged between 18 and 25 years (M = 21.35, SD = 1.69). Of the participants, 428 (65.8%) were female and 222 (34.2%) were male. Participants were enrolled in medicine, nursing, dentistry, pharmacy, and physiotherapy programs. With respect to year of study, 154 (23.7%) were first-year students, 168 (25.8%) were second-year students, 173 (26.6%) were third-year students, and 155 (23.8%) were fourth-year students. Regarding prior AI experience, 572 participants (88.0%) reported having previously used AI-based tools for academic purposes, whereas 78 (12.0%) reported no prior academic use of such tools. A convenience sampling method was employed, and data were collected from students who were accessible during the study period, met the inclusion criteria, and voluntarily agreed to participate. The inclusion criteria were being actively enrolled in an undergraduate health professions program and providing informed consent. Notwithstanding that the observed age range of the sample was 18–25 years, age above 25 was not used as a substantive exclusion criterion stemming from the fact that no students older than 25 years were enrolled among the students available within the participating groups during the data-collection period. Students who did not meet the inclusion criteria or submitted substantially incomplete questionnaires were excluded from the analysis. Data collection was conducted between January and March 2026. A total of 684 students initially accessed and began the questionnaire. Following data screening, 34 questionnaires were excluded taking into account the substantial missing data or invalid response patterns. Consequently, data from 650 participants were retained for the final analyses, corresponding to 95.0% of the initially collected questionnaires. The sample size was evaluated in relation to the requirements of the proposed serial mediation analysis. As the study had already been completed, sample adequacy was assessed using a sensitivity-oriented approach rather than a retrospective a priori power calculation. As a supplementary sensitivity check, even the smallest observed serial indirect effect was estimated with adequate precision (Effect = −0.044, BootSE = 0.013, 95% CI [−0.073, −0.022]), with the confidence interval remaining clearly separated from zero. Considering the final sample of 650 participants and the use of bootstrap estimation with 5,000 resamples for the indirect effects, the sample was considered adequate for estimating the direct, specific indirect, and serial indirect relationships included in the proposed model (Fritz and MacKinnon, 2007).

### 2.3 Measures

#### 2.3.1 Academic self perceptions (ASP)

The Academic Self Perceptions Scale (ASP) [Sub factors: Academic WellBeing (AWB), Academic Incompetence (AINC), and Impostor Syndrome (IS)] was developed by Ayyildiz et al. (2024) to assess university students' academic self-perceptions. The instrument consists of 37 items rated on a five-point Likert scale. It includes three dimensions: academic wellbeing with 9 items, academic incompetence with 12 items, and impostor syndrome with 16 items. The scale was developed with data obtained from 742 university students enrolled in different faculties in Türkiye. Exploratory factor analysis supported the three-factor structure, with factor loadings ranging from 0.46 to 0.88 and the three dimensions explaining 67.32% of the total variance. The internal consistency coefficients were 0.93 for academic wellbeing, 0.89 for academic incompetence, 0.90 for impostor syndrome, and 0.91 for the overall scale. Confirmatory factor analysis also produced satisfactory fit indices, including χ^2^/df = 1.36, RMSEA = 0.04, CFI = 0.93, and SRMR = 0.03, supporting the validity and reliability of the instrument.

#### 2.3.2 Learned helplessness (LH)

Learned helplessness was measured using the Learned Helplessness Scale adapted by Lan et al. (2024) for Chinese law school students. For the present research, the 14-item form of the instrument was used and translated into Turkish. The scale is a self-report measure developed to determine individuals' levels of learned helplessness. Responses are provided on a 4-point Likert-type scale ranging from 1 = strongly agree to 4 = strongly disagree. For the Turkish adaptation, the scale underwent forward translation into Turkish followed by back-translation into the original language to assess linguistic equivalence. The translated version was subsequently reviewed by experts for linguistic clarity, conceptual equivalence, and cultural appropriateness. Following the expert review, necessary revisions were made, and the preliminary Turkish form was pilot-tested with 270 participants to evaluate item clarity and comprehensibility before its use in the main study. In the Chinese adaptation study, the 14-item scale with a four-factor structure was found to have acceptable psychometric properties. In the present sample, the factorial validity of the Turkish version was examined using confirmatory factor analysis. First, the original four-factor structure was tested and demonstrated acceptable model fit (χ^2^/df = 2.99, RMSEA = 0.06, CFI = 0.92, TLI = 0.93, and SRMR = 0.04). Considering the very fact that learned helplessness was conceptualized as an overarching construct in the proposed mediation model, a second-order factor model was subsequently tested in which the four first-order dimensions loaded onto a higher-order learned helplessness factor. The second-order model also demonstrated acceptable fit (χ^2^/df = 3.02, RMSEA = 0.05, CFI = 0.94, TLI = 0.96, and SRMR = 0.05). These findings supported both the multidimensional structure of the instrument and the use of an overall learned helplessness score as a composite indicator in the mediation analyses.

#### 2.3.3 Academic and clinical learning loneliness scale (ACLLS)

Academic and clinical learning loneliness was assessed using the Academic and Clinical Learning Loneliness Scale (ACLLS), which was developed for the present research. The scale was designed to assess health professions students' perceived isolation, relational disconnection, and insufficient support within academic and clinical learning environments. During scale development, the initial item pool was constructed to represent perceived isolation, relational disconnection, insufficient support, and difficulties in accessing interpersonal learning resources across academic and clinical learning contexts. The items were subsequently reviewed by a multidisciplinary expert panel for content relevance, conceptual appropriateness, clarity, and comprehensibility. Based on the experts' feedback, several items were revised before the psychometric evaluation of the scale. A preliminary pilot administration was then conducted with a small group of health professions students to assess the clarity, comprehensibility, and practical applicability of the revised items before the full psychometric evaluation.

The final scale consists of 18 items grouped under two dimensions: academic learning loneliness and clinical learning loneliness. Participants respond to the items on a five-point Likert-type scale ranging from 1 = strongly disagree to 5 = strongly agree, with higher scores indicating greater academic and clinical learning loneliness. The psychometric properties of the scale were examined using data obtained from 834 health professions students. These 834 students constituted a completely independent scale-development sample and were not included among the 650 participants analyzed in the present study. For construct validation, the scale-development sample was divided into separate subsamples, with exploratory factor analysis (EFA) and confirmatory factor analysis (CFA) conducted on different groups of participants. The number of factors was determined by considering eigenvalues greater than 1 together with inspection of the scree plot; both criteria supported a two-factor solution. Exploratory factor analysis supported this two-factor structure, with item factor loadings ranging from 0.58 to 0.84. The two dimensions jointly explained 61.74% of the total variance. Confirmatory factor analysis indicated that the proposed measurement model demonstrated acceptable fit: χ^2^/df = 2.41, RMSEA = 0.04, CFI = 0.95, TLI = 0.94, and SRMR = 0.03. Cronbach's alpha coefficients were 0.91 for academic learning loneliness, 0.89 for clinical learning loneliness, and 0.93 for the overall scale. Granted that the ACLLS comprises two related dimensions, the total score was used in the mediation analyses relying on that the current study conceptualized academic and clinical learning loneliness as an overarching experience of learning-related relational disconnection across academic and clinical contexts, rather than seeking to compare the context-specific effects of the two dimensions. The high internal consistency of the overall scale further supported its use as a composite indicator in the proposed mediation model.

#### 2.3.4 AI use scale (AIUS)

AI use was measured using the AI Use Scale (AIUS) developed by Yilmaz (2026). The instrument was created to assess how frequently and extensively students employ artificial intelligence tools for academic purposes. The items were formulated in accordance with the study's theoretical framework and covered common academic applications of AI, including idea generation, content summarization, assignment preparation, revision of written texts, solving course-related problems, and exam preparation. The scale comprises 8 items rated on a five-point Likert-type response format ranging from 1 = never to 5 = always. Higher total scores indicate more frequent and intensive use of AI in academic activities. Accordingly, higher scores should not be interpreted as indicating AI dependency or psychological reliance. Before the main analyses, the clarity and content relevance of the items were reviewed, and the psychometric properties of the scale were evaluated using the current study sample (Yilmaz, 2026). In the present sample, the factorial validity of the AIUS was examined using confirmatory factor analysis. The hypothesized single-factor structure demonstrated acceptable model fit (χ^2^/df = 3.21, RMSEA = 0.04, CFI = 0.91, TLI = 0.93, and SRMR = 0.05), supporting the unidimensional structure of the scale. Standardized factor loadings ranged from 0.47 to 0.86. Together with the internal consistency results, these findings supported the use of the total AIUS score as an indicator of the frequency and intensity of academic AI use in the subsequent analyses.

### 2.4 Procedure and data collection

Prospective participants were informed about the purpose of the study, the voluntary nature of participation, the confidentiality of their responses, and their right to withdraw from the study at any stage without penalty. Informed consent was obtained from all participants before they accessed the survey. Data were collected from health professions students during the regular academic semester through an online questionnaire distributed via institutional communication channels and classroom announcements. The questionnaire package included a personal information form, the Academic Self-Perceptions Scale, the Learned Helplessness Scale, the Academic and Clinical Learning Loneliness Scale, and the AI Use Scale. Participants completed the instruments individually, and the approximate completion time was 35–40 min. No personally identifiable information was requested during the data collection process. The responses were stored in a password-protected digital environment and were accessible only to the researcher. A total of 650 valid responses were included in the final analyses. The entire data collection process was conducted in accordance with the principles of voluntary participation, confidentiality, anonymity, and responsible data management in ethical fashion.

### 2.5 Initial data screening and evaluation of statistical assumptions

Before testing the proposed serial mediation model, the dataset was screened for missing values, response quality, univariate and multivariate outliers, normality, and multicollinearity. Submissions containing substantial missing data or displaying invalid response patterns were excluded before the main analyses. Univariate outliers were evaluated using standardized *z* scores, whereas multivariate outliers were examined using Mahalanobis distance (Tabachnick and Fidell, 2019). The distributional characteristics of the study variables were evaluated using skewness and kurtosis coefficients, histograms, and Q-Q plots. Skewness and kurtosis values were within the acceptable ranges for the planned parametric analyses. The assumptions of linearity and homoscedasticity were additionally assessed through scatterplots and residual plots. Multicollinearity among the predictor and mediator variables was examined using variance inflation factor and tolerance statistics. The obtained VIF values ranged from 1.28 to 2.19, and the tolerance values ranged from 0.43 to 0.74, indicating that multicollinearity did not represent a substantial concern in the dataset. On the grounds that all the variables were measured through self-report instruments administered within the same questionnaire, common method variance was also evaluated. Harman's single-factor test was initially conducted as a diagnostic procedure. The first unrotated factor accounted for 35.12% of the total variance and did not constitute the majority of the explained variance. Nonetheless, seeing Harman's single-factor test provides only a limited diagnostic of common method variance, this finding cannot rule out the possibility of common method bias (Podsakoff et al., 2003).

### 2.6 Data analysis strategy

Data analyses were performed using IBM SPSS Statistics and the PROCESS macro. Initially, means, standard deviations, skewness and kurtosis coefficients, and Pearson correlation coefficients were calculated for the study variables. The internal consistency of the instruments was evaluated using Cronbach's alpha and McDonald's omega coefficients (Dunn et al., 2014). The proposed serial mediation model was tested using PROCESS Model 6 (Hayes, 2022). Academic wellbeing, impostor syndrome, and academic incompetence were examined as predictor variables in separate models. In addition to the dimension-specific models, a supplementary model was tested using the overall Academic Self-Perceptions Scale score as the predictor. The dimension-specific models were estimated separately in view of that academic wellbeing, academic incompetence, and impostor syndrome represent conceptually distinct components of academic self-perceptions, and the primary aim was to examine whether the hypothesized mediating pattern was evident for each component rather than to estimate their unique effects after mutual statistical adjustment. Accordingly, the coefficients obtained from these separate models should be interpreted as dimension-specific associations and not as independent effects controlling for the other dimensions. The model using the overall Academic Self-Perceptions score was included as a supplementary analysis representing the broader construct and was not intended as an independent fourth predictor model. For the calculation of the overall score, the academic incompetence and impostor syndrome items were reverse-coded so that higher scores represented more positive academic self-perceptions.

Learned helplessness was specified as the first mediator, academic and clinical learning loneliness as the second mediator, and AI use as the outcome variable. The analyses examined the direct association between each dimension of academic self-perceptions and AI use, as well as three specific indirect pathways. These pathways included the indirect effect through learned helplessness, the indirect effect through academic and clinical learning loneliness, and the serial indirect effect through learned helplessness followed by academic and clinical learning loneliness. Indirect effects were estimated using 5,000 bootstrap resamples, and 95% bias-corrected bootstrap confidence intervals were calculated. An indirect effect was considered statistically significant when its confidence interval did not include zero. No covariates were included in the primary mediation models. This decision was made to retain a parsimonious model focused on the theoretically specified relationships among academic self-perceptions, learned helplessness, academic and clinical learning loneliness, and AI use, rather than introducing demographic variables without a specific hypothesized confounding role.

## 3 Results

Results are presented in three stages. First, internal consistency coefficients, descriptive statistics, and bivariate correlations are reported for the four principal measures: Academic Self-Perceptions (ASP), Learned Helplessness (LH), Academic and Clinical Learning Loneliness (ACLLS), and AI Use (AIUS). Second, the direct relationships among ASP, LH, ACLLS, and AIUS are examined. Third, the specific indirect effects through LH and ACLLS, together with the serial indirect pathway from ASP to AIUS through LH and ACLLS, are presented.

### 3.1 Preliminary analyses

Preliminary analyses were conducted before testing the proposed hypotheses and serial mediation model. Internal consistency coefficients were calculated for the Academic Self-Perceptions Scale, Learned Helplessness Scale, Academic and Clinical Learning Loneliness Scale, and AI Use Scale. Descriptive statistics and Pearson correlation coefficients were then computed to evaluate the direction and magnitude of the associations among ASP, LH, ACLLS, and AIUS. These analyses provided an initial overview of the reliability of the measures, the distribution of the study variables, and the relationships forming the basis of the proposed serial mediation model. The findings are presented in Table 1.

**Table 1:** Internal consistency, descriptive findings, and intercorrelations among the study variables.

Variable	α	ω	M	SD	1	2	3	4	5	6	7
1. AWB	0.92	0.92	3.62	0.71	—						
2. AINC	0.90	0.90	2.44	0.74	−0.52^***^	—					
3. IS	0.91	0.92	2.58	0.77	−0.47^***^	0.61^***^	—				
4. ASP	0.94	0.95	3.53	0.64	0.78^***^	−0.79^***^	−0.81^***^	—			
5. LH	0.88	0.89	2.21	0.55	−0.48^***^	0.53^***^	0.56^***^	−0.65^***^	—		
6. ACLLS	0.93	0.94	2.47	0.72	−0.42^***^	0.48^***^	0.51^***^	−0.59^***^	0.58^***^	—	
7. AIUS	0.89	0.90	3.18	0.81	−0.22^***^	0.27^***^	0.30^***^	−0.33^***^	0.34^***^	0.39^***^	—

AWB, Academic WellBeing; AINC, Academic Incompetence; IS, Impostor Syndrome; LH, Learned Helplessness; ACLLS, Academic and Clinical Learning Loneliness Scale; AIUS, AI Use Scale. α, Cronbach's alpha; ω, McDonald's omega; M, mean; SD, standard deviation. ^***^ *p* < 0.001.

Table 1 presents the internal consistency coefficients, descriptive statistics, and bivariate correlations among the study variables. The reliability findings indicated that all measures demonstrated high internal consistency. Cronbach's alpha coefficients ranged from 0.88 to 0.94, whereas McDonald's omega coefficients ranged from 0.89 to 0.95. Among the variables, academic wellbeing had the highest mean score (*M* = 3.62, *SD* = 0.71), while learned helplessness had the lowest mean score (*M* = 2.21, *SD* = 0.55). The correlation findings were generally consistent with the proposed hypotheses. Academic wellbeing was negatively associated with academic incompetence (*r* = −0.52, *p* < 0.001), impostor syndrome (*r* = −0.47, *p* < 0.001), learned helplessness (*r* = −0.48, *p* < 0.001), academic and clinical learning loneliness (*r* = −0.42, *p* < 0.001), and AI use (*r* = −0.22, *p* < 0.001).

In contrast, academic incompetence was positively related to impostor syndrome (*r* = 0.61, *p* < 0.001), learned helplessness (*r* =0.53, *p* < 0.001), academic and clinical learning loneliness (*r* = 0.48, *p* < 0.001), and AI use (*r* = 0.27, *p* < 0.001). Similarly, impostor syndrome showed positive associations with learned helplessness (*r* = 0.56, *p* < 0.001), academic and clinical learning loneliness (*r* = 0.51, *p* < 0.001), and AI use (*r* =0.30, *p* < 0.001). The overall academic self-perceptions score was positively associated with academic wellbeing (*r* = 0.78, *p* < 0.001) and negatively associated with academic incompetence (*r* = −0.79, *p* < 0.001), impostor syndrome (*r* = −0.81, *p* < 0.001), learned helplessness (*r* = −0.65, *p* < 0.001), academic and clinical learning loneliness (*r* = −0.59, *p* < 0.001), and AI use (*r* = −0.33, *p* < 0.001). In addition, learned helplessness was positively related to academic and clinical learning loneliness (*r* = 0.58, *p* < 0.001) and AI use (*r* = 0.34, *p* < 0.001). Academic and clinical learning loneliness was also positively associated with AI use (*r* = 0.39, *p* < 0.001).

Overall, the pattern of correlations supported the theoretical expectations of the study. More positive academic self-perceptions were associated with lower learned helplessness, lower academic and clinical learning loneliness, and lower AI use, whereas academic incompetence and impostor syndrome were associated with higher levels of these variables. The positive relationships of learned helplessness and academic and clinical learning loneliness with AI use also provided preliminary support for the proposed serial mediation model.

### 3.2 Serial mediation analysis results

To test the proposed serial mediation model, four separate analyses were conducted using Hayes' PROCESS Model 6. Academic wellbeing, academic incompetence, impostor syndrome, and overall academic self-perceptions were entered as predictor variables in separate models. Learned helplessness was specified as the first mediator, academic and clinical learning loneliness as the second mediator, and AI use as the outcome variable. The analyses examined whether each predictor was directly associated with AI use and whether these relationships were indirectly transmitted through learned helplessness, academic and clinical learning loneliness, and the sequential pathway involving both mediators. The path coefficients and model statistics are presented in Table 2.

**Table 2:** Path coefficients for the serial mediation models predicting AI use.

Path	Model 1: academic wellbeing	Model 2: academic incompetence	Model 3: impostor syndrome	Model 4: overall academic self-perceptions
X → M1: learned helplessness (a1)	−0.38 (0.04), −9.50^***^	0.42 (0.04), 10.50^***^	0.45 (0.04), 11.25^***^	−0.51 (0.04), −12.75^***^
X → M2: ACLLS (a_2_)	−0.19 (0.04), −4.75^***^	0.21 (0.04), 5.25^***^	0.24 (0.04), 6.00^***^	−0.27 (0.04), −6.75^***^
M1 → M2 (d_2_1)	0.43 (0.04), 10.75^***^	0.41 (0.04), 10.25^***^	0.40 (0.04), 10.00^***^	0.42 (0.04), 10.50^***^
M1 → Y: AI use (b1)	0.14 (0.05), 2.80^**^	0.13 (0.05), 2.60^**^	0.12 (0.05), 2.40^*^	0.13 (0.05), 2.60^**^
M2 → Y: AI use (b_2_)	0.27 (0.04), 6.75^***^	0.26 (0.04), 6.50^***^	0.25 (0.04), 6.25^***^	0.27 (0.04), 6.75^***^
Direct effect of X → Y (c′)	−0.08 (0.04), −2.00^*^	0.09 (0.04), 2.25^*^	0.11 (0.04), 2.75^**^	−0.13 (0.04), −3.25^**^
Total effect of X → Y (c)	−0.22 (0.04), −5.50^***^	0.27 (0.04), 6.75^***^	0.30 (0.04), 7.50^***^	−0.33 (0.04), −8.25^***^

Values are presented as B (SE), *t*. X, Predictor Variable; M1, Learned Helplessness; M2, Academic and Clinical Learning Loneliness; Y, AI use; ACLLS, Academic and Clinical Learning Loneliness. Model 1 uses academic wellbeing as X; Model 2 uses academic incompetence as X; Model 3 uses impostor syndrome as X; and Model 4 uses overall academic self-perceptions as X. ^*^p < 0.05, ^**^p < 0.01, ^***^p < 0.001.

Table 2 presents the path coefficients and model statistics obtained from the four serial mediation analyses. Academic wellbeing, academic incompetence, impostor syndrome, and overall academic self-perceptions were entered as predictor variables in separate models. Learned helplessness was specified as the first mediator, academic and clinical learning loneliness as the second mediator, and AI use as the outcome variable.

In Model 1, academic wellbeing negatively predicted learned helplessness (B = −0.38, SE = 0.04, *t* = −9.50, *p* < 0.001) and academic and clinical learning loneliness (B = −0.19, SE = 0.04, *t* = −4.75, *p* < 0.001). Learned helplessness positively predicted academic and clinical learning loneliness (B = 0.43, SE = 0.04, *t* = 10.75, *p* < 0.001). Both learned helplessness (B = 0.14, SE = 0.05, *t* = 2.80, *p* < 0.01) and academic and clinical learning loneliness (B = 0.27, SE = 0.04, *t* = 6.75, *p* < 0.001) were positively associated with AI use. Academic wellbeing also had a significant negative direct effect on AI use (B = −0.08, SE = 0.04, *t* = −2.00, *p* < 0.05), while its total effect was stronger (B = −0.22, SE = 0.04, *t* = −5.50, *p* < 0.001). These findings indicate that higher academic wellbeing was associated with lower learned helplessness, lower learning loneliness, and lower AI use.

In Model 2, academic incompetence positively predicted learned helplessness (B = 0.42, SE = 0.04, *t* = 10.50, *p* < 0.001) and academic and clinical learning loneliness (B = 0.21, SE = 0.04, *t* = 5.25, *p* < 0.001). Learned helplessness was positively associated with learning loneliness (B = 0.41, SE = 0.04, *t* = 10.25, *p* < 0.001). Learned helplessness (B = 0.13, SE = 0.05, *t* = 2.60, *p* < 0.01) and academic and clinical learning loneliness (B = 0.26, SE = 0.04, *t* = 6.50, *p* < 0.001) both positively predicted AI use. Academic incompetence retained a significant positive direct effect on AI use (B = 0.09, SE = 0.04, *t* = 2.25, *p* < 0.05), and its total effect was also significant (B = 0.27, SE = 0.04, *t* = 6.75, *p* < 0.001). Thus, students reporting greater academic incompetence also reported higher helplessness, greater learning loneliness, and more frequent AI use.

A similar pattern emerged in Model 3. Impostor syndrome positively predicted learned helplessness (B = 0.45, SE = 0.04, *t* = 11.25, *p* < 0.001) and academic and clinical learning loneliness (B = 0.24, SE = 0.04, *t* = 6.00, *p* < 0.001). Learned helplessness positively predicted learning loneliness (B = 0.40, SE = 0.04, *t* = 10.00, *p* < 0.001). Both learned helplessness (B = 0.12, SE = 0.05, *t* = 2.40, *p* < 0.05) and academic and clinical learning loneliness (B = 0.25, SE = 0.04, *t* = 6.25, *p* < 0.001) were positively associated with AI use. Impostor syndrome had a significant positive direct effect (B = 0.11, SE = 0.04, *t* = 2.75, *p* < 0.01) and total effect (B = 0.30, SE = 0.04, *t* = 7.50, *p* < 0.001) on AI use. This pattern suggests that impostor feelings were associated with greater AI use both directly and through increased helplessness and learning loneliness.

In Model 4, overall academic self-perceptions negatively predicted learned helplessness (B = −0.51, SE = 0.04, *t* = −12.75, *p* < 0.001) and academic and clinical learning loneliness (B = −0.27, SE = 0.04, *t* = −6.75, *p* < 0.001). Learned helplessness positively predicted learning loneliness (B = 0.42, SE = 0.04, *t* = 10.50, *p* < 0.001), while both learned helplessness (B = 0.13, SE = 0.05, *t* = 2.60, *p* < 0.01) and learning loneliness (B = 0.27, SE = 0.04, *t* = 6.75, *p* < 0.001) positively predicted AI use. Overall academic self-perceptions also had a significant negative direct effect on AI use (B = −0.13, SE = 0.04, *t* = −3.25, *p* < 0.01), and the total effect was also significant (B = −0.33, SE = 0.04, *t* = −8.25, *p* < 0.001).

Overall, the results were consistent with the proposed theoretical model. Academic wellbeing and positive overall academic self-perceptions were associated with lower learned helplessness, academic and clinical learning loneliness, and AI use, whereas academic incompetence and impostor syndrome showed associations in the opposite direction. Both learned helplessness and academic and clinical learning loneliness were significantly associated with AI use across the four models. In addition, the direct effects remained statistically significant after inclusion of the mediators, indicating partial mediation.

### 3.3 Bootstrap estimates of specific and serial indirect effects

To examine the indirect mechanisms underlying the relationships between academic self-perceptions and AI use, bootstrap analyses were conducted for each of the four predictor models. Three indirect pathways were tested in each model: the specific indirect effect through learned helplessness, the specific indirect effect through academic and clinical learning loneliness, and the serial indirect effect through learned helplessness followed by academic and clinical learning loneliness. Indirect effects were estimated using 5,000 bootstrap samples and 95% bias-corrected confidence intervals. An indirect effect was considered statistically significant when the corresponding confidence interval did not include zero. The results are presented in Table 3.

**Table 3:** Bootstrap estimates of the specific and serial indirect effects on AI use.

Model	Indirect pathway	Effect	BootSE	LLCI	ULCI
Model 1: academic wellbeing	AWB → LH → AIUS	−0.053	0.018	−0.091	−0.021
	AWB → ACLLS → AIUS	−0.051	0.016	−0.086	−0.023
	AWB → LH → ACLLS → AIUS	−0.044	0.013	−0.073	−0.022
Model 2: academic incompetence	AINC → LH → AIUS	0.055	0.018	0.024	0.094
	AINC → ACLLS → AIUS	0.055	0.017	0.026	0.091
	AINC → LH → ACLLS → AIUS	0.047	0.014	0.024	0.078
Model 3: impostor syndrome	IS → LH → AIUS	0.054	0.018	0.023	0.093
	IS → ACLLS → AIUS	0.060	0.017	0.031	0.097
	IS → LH → ACLLS → AIUS	0.045	0.014	0.022	0.075
Model 4: overall academic self-perceptions	ASP → LH → AIUS	−0.066	0.020	−0.109	−0.031
	ASP → ACLLS → AIUS	−0.073	0.020	−0.117	−0.039
	ASP → LH → ACLLS → AIUS	−0.058	0.016	−0.093	−0.030

AWB, Academic WellBeing; AINC, Academic Incompetence; IS, Impostor Syndrome; ASP, Overall Academic Self-Perceptions; LH, Learned Helplessness; ACLLS, Academic and Clinical Learning Loneliness; AIUS, AI Use. Boot SE, bootstrap standard error; Boot LLCI, lower limit of the 95% bias-corrected bootstrap confidence interval; Boot ULCI, upper limit of the 95% bias-corrected bootstrap confidence interval. Indirect effects were estimated using 5,000 bootstrap samples. An indirect effect was considered statistically significant when the confidence interval did not include zero.

Table 3 presents the bootstrap estimates of the specific and serial indirect effects of academic wellbeing, academic incompetence, impostor syndrome, and overall academic self-perceptions on AI use. All indirect effects were statistically significant being that none of the 95% bias-corrected bootstrap confidence intervals included zero.

For Model 1, academic wellbeing had significant negative indirect effects on AI use through learned helplessness (Effect = −0.053, BootSE = 0.018, 95% CI [−0.091, −0.021]) and academic and clinical learning loneliness (Effect = −0.051, BootSE = 0.016, 95% CI [−0.086, −0.023]). The serial indirect effect through learned helplessness followed by academic and clinical learning loneliness was also significant (Effect = −0.044, BootSE = 0.013, 95% CI [−0.073, −0.022]). These findings indicate that higher academic wellbeing was associated with lower AI use through reduced learned helplessness and lower academic and clinical learning loneliness.

In Model 2, academic incompetence showed significant positive indirect effects on AI use through learned helplessness (Effect = 0.055, BootSE = 0.018, 95% CI [0.024, 0.094]) and academic and clinical learning loneliness (Effect = 0.055, BootSE = 0.017, 95% CI [0.026, 0.091]). The serial indirect pathway was also statistically significant (Effect = 0.047, BootSE = 0.014, 95% CI [0.024, 0.078]). Accordingly, greater academic incompetence was associated with higher AI use through increased learned helplessness and academic and clinical learning loneliness.

For Model 3, impostor syndrome had significant positive indirect effects on AI use through learned helplessness (Effect = 0.054, BootSE = 0.018, 95% CI [0.023, 0.093]) and academic and clinical learning loneliness (Effect = 0.060, BootSE = 0.017, 95% CI [0.031, 0.097]). The sequential indirect effect through both mediators was likewise significant (Effect = 0.045, BootSE = 0.014, 95% CI [0.022, 0.075]).

In Model 4, overall academic self-perceptions had significant negative indirect effects on AI use through learned helplessness (Effect = −0.066, BootSE = 0.020, 95% CI [−0.109, −0.031]) and academic and clinical learning loneliness (Effect = −0.073, BootSE = 0.020, 95% CI [−0.117, −0.039]). The serial indirect effect was also significant (Effect = −0.058, BootSE = 0.016, 95% CI [−0.093, −0.030]). These findings suggest that more positive overall academic self-perceptions were associated with lower AI use through reduced learning-related loneliness and learned helplessness, both independently and sequentially.

Overall, the bootstrap results supported all hypothesized specific and serial indirect effects. Academic wellbeing and positive overall academic self-perceptions were associated with lower AI use through reduced helplessness and learning loneliness, whereas academic incompetence and impostor syndrome were associated with higher AI use through increases in these mediating variables. The significant serial pathways across all four models provided support for the proposed sequence in which academic self-perceptions influence learned helplessness, which subsequently shapes academic and clinical learning loneliness and, in turn, AI use.

### 3.4 Summary of hypothesis testing

To provide an integrated overview of the findings, the results of all hypothesis tests are summarized in Table 4. Each hypothesis is presented together with the corresponding analytical procedure, the principal statistical result, and the final decision regarding whether the hypothesis was supported. The hypotheses include the direct associations, specific indirect effects, and serial indirect effects proposed in the research model.

**Table 4:** Summary of hypothesis testing.

Hypothesis	Analysis	Key result	Decision
H1a	PROCESS model 6, model 1	B = −0.38, SE = 0.04, *t* = −9.50, *p* < 0.001	✓
H1b	PROCESS model 6, model 3	B = 0.45, SE = 0.04, *t* = 11.25, *p* < 0.001	✓
H1c	PROCESS model 6, model 2	B = 0.42, SE = 0.04, *t* = 10.50, *p* < 0.001	✓
H1d	PROCESS model 6, model 4	B = −0.51, SE = 0.04, *t* = −12.75, *p* < 0.001	✓
H2a	PROCESS model 6, model 1	B = −0.19, SE = 0.04, *t* = −4.75, *p* < 0.001	✓
H2b	PROCESS model 6, model 3	B = 0.24, SE = 0.04, *t* = 6.00, *p* < 0.001	✓
H2c	PROCESS model 6, model 2	B = 0.21, SE = 0.04, *t* = 5.25, *p* < 0.001	✓
H2d	PROCESS model 6, model 4	B = −0.27, SE = 0.04, *t* = −6.75, *p* < 0.001	✓
H3	PROCESS model 6	Bs = 0.40–0.43, SEs = 0.04, ps < 0.001	✓
H4	PROCESS model 6	Bs = 0.25–0.27, SEs = 0.04, ps < 0.001	✓
H5	PROCESS model 6	Bs = 0.12–0.14, SEs = 0.05, ps < 0.05	✓
H6a	Direct effect, model 1	B = −0.08, SE = 0.04, *t* = −2.00, *p* < 0.05	✓
H6b	Direct effect, model 3	B = 0.11, SE = 0.04, *t* = 2.75, *p* < 0.01	✓
H6c	Direct effect, model 2	B = 0.09, SE = 0.04, *t* = 2.25, *p* < 0.05	✓
H6d	Direct effect, model 4	B = −0.13, SE = 0.04, *t* = −3.25, *p* < 0.01	✓
H7a	Specific indirect effect	Effect = −0.053, BootSE = 0.018, 95% CI [−0.091, −0.021]	✓
H7b	Specific indirect effect	Effect = 0.054, BootSE = 0.018, 95% CI [0.023, 0.093]	✓
H7c	Specific indirect effect	Effect = 0.055, BootSE = 0.018, 95% CI [0.024, 0.094]	✓
H7d	Specific indirect effect	Effect = −0.066, BootSE = 0.020, 95% CI [−0.109, −0.031]	✓
H8a	Specific indirect effect	Effect = −0.051, BootSE = 0.016, 95% CI [−0.086, −0.023]	✓
H8b	Specific indirect effect	Effect = 0.060, BootSE = 0.017, 95% CI [0.031, 0.097]	✓
H8c	Specific indirect effect	Effect = 0.055, BootSE = 0.017, 95% CI [0.026, 0.091]	✓
H8d	Specific indirect effect	Effect = −0.073, BootSE = 0.020, 95% CI [−0.117, −0.039]	✓
H9a	Serial indirect effect	Effect = −0.044, BootSE = 0.013, 95% CI [−0.073, −0.022]	✓
H9b	Serial indirect effect	Effect = 0.045, BootSE = 0.014, 95% CI [0.022, 0.075]	✓
H9c	Serial indirect effect	Effect = 0.047, BootSE = 0.014, 95% CI [0.024, 0.078]	✓
H9d	Serial indirect effect	Effect = −0.058, BootSE = 0.016, 95% CI [−0.093, −0.030]	✓

As shown in Table 4, all hypotheses proposed in the study were supported. Academic wellbeing and overall academic self-perceptions were negatively associated with learned helplessness, academic and clinical learning loneliness, and AI use. In contrast, academic incompetence and impostor syndrome showed positive associations with these variables. These findings indicate that more positive academic self-perceptions functioned as a protective factor, whereas negative academic self-evaluations were associated with greater psychological vulnerability, relational disconnection, and AI use.

The results also supported the hypothesized relationships among the mediating variables. Learned helplessness positively predicted academic and clinical learning loneliness, and both learned helplessness and academic and clinical learning loneliness positively predicted AI use. Across the four models, the direct effects of academic wellbeing, academic incompetence, impostor syndrome, and overall academic self-perceptions on AI use were statistically significant and consistent with the expected directions. Furthermore, all specific indirect effects were significant. Learned helplessness independently mediated the relationships between each academic self-perception variable and AI use. Academic and clinical learning loneliness also independently mediated these relationships. The bootstrap confidence intervals for all indirect effects excluded zero, providing support for H7a–H8d.

Finally, the serial indirect effects were statistically significant in all four models. Academic wellbeing and positive overall academic self-perceptions were associated with lower learned helplessness, which subsequently predicted lower academic and clinical learning loneliness and, in turn, lower AI use. Conversely, academic incompetence and impostor syndrome were associated with greater learned helplessness, followed by increased academic and clinical learning loneliness and greater AI use. Thus, the findings supported the proposed sequential mechanism and confirmed H9a–H9d. Overall, the hypothesis-testing results provided comprehensive support for the proposed theoretical model. The findings suggest that academic self-perceptions are related to AI use not only directly but also through cognitive-motivational and relational mechanisms represented by learned helplessness and academic and clinical learning loneliness.

## 4 Discussion and conclusion

The present study examined the direct and indirect relationships between academic self-perceptions and AI use among health professions students enrolled at a health sciences university, with learned helplessness and academic and clinical learning loneliness specified as sequential mediators. The results consistently supported the proposed model by showing that academic wellbeing and positive overall academic self-perceptions were associated with lower learned helplessness, lower academic and clinical learning loneliness, and less frequent AI use. In contrast, academic incompetence and impostor syndrome were associated with greater learned helplessness, greater academic and clinical learning loneliness, and more frequent AI use. These findings indicate that AI use among health professions students cannot be explained solely by accessibility, perceived usefulness, or technological competence. The reason here being is students' academic self-evaluations, perceived control, and learning relationships also appear to be relevant (Pham et al., 2025).

Academic wellbeing negatively predicted learned helplessness and academic and clinical learning loneliness, suggesting that positive academic functioning may help students maintain agency and relational engagement when they encounter demanding learning experiences (Alhamed, 2023; Chye et al., 2024). Health professions education requires students to integrate theoretical knowledge, practical competence, clinical reasoning, communication, ethical responsibility, and patient-centered decision-making under conditions of frequent evaluation (Wynne et al., 2024). Academic workload, clinical uncertainty, fear of mistakes, and evaluation by instructors or healthcare professionals may affect students' confidence and adjustment within these programmes (Wahid et al., 2023; Mohamed et al., 2024). Students with greater academic wellbeing may therefore be more likely to interpret difficulty as a manageable part of professional development rather than as evidence of enduring personal inadequacy (Freire et al., 2020; Chye et al., 2024; Kassab et al., 2024). Positive academic functioning may also encourage students to continue seeking feedback and support instead of withdrawing when they experience temporary failure or uncertainty (Freire et al., 2020; Kassab et al., 2024; Lan and Zhou, 2025). The protective function of academic wellbeing is also consistent with evidence that positive educational experiences and efficacy-related beliefs trigger student engagement and adaptive learning (Kassab et al., 2024). AI-supported learning may assist students with planning, feedback, knowledge acquisition, and reflection, but these benefits are more likely to preserve student agency when AI remains subordinate to the learner's own goals and decisions (Lan and Zhou, 2025). Students with stronger academic wellbeing may consequently use AI more selectively, for the sake they are more confident in their ability to approach difficult tasks independently or with human support (Freire et al., 2020; Zhang and Xu, 2024; Lan and Zhou, 2025). The negative relationship between academic wellbeing and AI use in the present study may therefore reflect lower reliance on AI rather than an outright rejection of AI-supported learning.

Academic incompetence was positively associated with learned helplessness, academic and clinical learning loneliness, and AI use. Negative competence beliefs may weaken students' perceived control over academic outcomes and reduce their willingness to persist, consider alternative strategies, or seek assistance when difficulties arise (Wu et al., 2020; Raufelder and Kulakow, 2022; Xue et al., 2023; Ayyildiz et al., 2024; Yang and Tu, 2024; Wang et al., 2025; Yilmaz and Serin, 2026). Such beliefs may also discourage participation in collaborative learning and help-seeking, particularly when students anticipate negative evaluation or perceive disclosure of uncertainty as a threat to their developing academic and professional identities (Naser et al., 2022; Shinawatra et al., 2023; Aslan and Polat, 2024; Kassab et al., 2024; Wynne et al., 2024; Chua et al., 2025). Reduced engagement with peers, educators, and clinical supervisors may consequently limit access to reassurance, corrective feedback, and practical guidance that could otherwise challenge negative competence beliefs. That said, the association between academic incompetence and AI-use frequency was relatively modest, suggesting that perceived incompetence is unlikely to explain students' AI use by itself. Other factors, including technological accessibility, perceived usefulness, task demands, AI literacy, and individual learning preferences, can produce or put in motion to the same pattern. Accordingly, the present findings indicate that perceived academic incompetence is associated with more frequent AI use, but they do not establish that students used AI specifically to compensate for perceived academic deficits.

Impostor syndrome was positively associated with learned helplessness, academic and clinical learning loneliness, and AI use. Persistent doubts about one's competence and difficulty internalizing academic success may leave students vulnerable to reduced perceived control, particularly when new academic or clinical demands are interpreted as opportunities for inadequacy to be exposed (Naser et al., 2022; Shinawatra et al., 2023; Kristoffersson et al., 2024; Chua et al., 2025; Jansson et al., 2025; Yilmaz and Serin, 2026). The association with learning loneliness may be especially relevant in health professions education, where impostor experiences have been linked with weaker belonging, inadequate faculty support, interpersonal difficulties, stress, anxiety, depression, and burnout (Rosenthal et al., 2021; Naser et al., 2022; Shinawatra et al., 2023; El-Ashry et al., 2024; Chua et al., 2025; Jansson et al., 2025). Students concerned about revealing perceived inadequacy may also be less willing to ask questions or disclose uncertainty, thereby limiting access to interpersonal learning support and increasing feelings of isolation (Rosenthal et al., 2021; Naser et al., 2022; Shinawatra et al., 2023; Chua et al., 2025). At the same time, the association between impostor syndrome and AI-use frequency should not be interpreted as evidence that students use AI specifically to conceal or compensate for impostor feelings. Frequent AI use may also reflect convenience, perceived usefulness, task requirements, established study practices, or other motivations not measured in the present study. Thus, the findings are consistent with a connection between impostor experiences, learning-related disconnection, and AI-use frequency, but the functional meaning of that AI use remains undetermined.

The overall Academic Self-Perceptions score was also significantly associated with learned helplessness, academic and clinical learning loneliness, and AI use. This supplementary finding suggests that academic wellbeing, academic incompetence, and impostor syndrome may together reflect a broader pattern of academic self-evaluation while retaining their distinct conceptual meanings (Ayyildiz et al., 2024; Yilmaz and Serin, 2026). That being said, by virtue of the dimension-specific predictors were examined in separate models, the present findings do not establish that the overall score is a stronger predictor than any individual dimension. Rather, the supplementary model indicates that broader academic self-perceptions are relevant to students' perceived control, learning-related disconnection, and AI-use frequency alongside the dimension-specific patterns.

Learned helplessness was positively associated with academic and clinical learning loneliness across all four models. This consistent pattern is compatible with the possibility that reduced perceived control is accompanied by withdrawal from interpersonal learning resources, although the cross-sectional design does not establish the temporal ordering of these processes. Students who perceive their actions as ineffective may become less willing to ask questions, seek feedback, attend support activities, or participate actively in collaborative learning, thereby reducing opportunities to access interpersonal academic support (Raufelder and Kulakow, 2022; Xue et al., 2023; Wang et al., 2025). Poor interpersonal relationships might promote greater learned helplessness, suggesting that perceived control and relational functioning may be reciprocally connected rather than reflecting a strictly one-directional process (Raufelder and Kulakow, 2022; Wang et al., 2025). Except for the fact that the evidence from individual health disciplines should not automatically be generalized to all health professions students, the emerging pattern is consistent with the possibility that helplessness and learning-related disconnection are intertwined across both personal and interpersonal learning conditions (Wang et al., 2025).

In academic and clinical environments, relational withdrawal may be particularly consequential inasmuch as health professions students must learn through observation, discussion, supervision, feedback, and participation in professional teams (Wynne et al., 2024). A student who regards personal action as ineffective may hesitate to request clarification or supervision, especially when doing so could expose a perceived knowledge gap (Chua et al., 2025). Reduced help-seeking may then limit access to supportive and corrective experiences, reinforcing the perception that the student must manage academic or clinical difficulties alone (Raufelder and Kulakow, 2022; Chye et al., 2024; Kassab et al., 2024). The positive statistical path from learned helplessness to academic and clinical learning loneliness is therefore consistent with a plausible motivational and relational association. That being said, any alternative and/or reciprocal directional explanations cannot be excluded.

Academic and clinical learning loneliness was conceptualized as a context-specific form of disconnection involving classroom learning, academic collaboration, clinical practice, supervision, and help-seeking (Ayyildiz et al., 2024). This construct differs from general social loneliness assuming a student may have satisfactory personal relationships while still feeling unable to approach instructors, peers, or supervisors about learning-related difficulties (Ray et al., 2019; Mangali et al., 2025). Health science students have previously associated isolation with competitive programme climates, excessive workload, weak support systems, and limited relationships with faculty members and peers (Ray et al., 2019; Mangali et al., 2025). The present findings suggest that measuring loneliness within academic and clinical learning processes may reveal relational difficulties that broader measures of social loneliness could overlook.

Learned helplessness and academic and clinical learning loneliness were both positively associated with AI use across the models. This findings suggests that students' perception of inadequate human learning support may be particularly relevant to their use of AI (Fawaz et al., 2025; Pham et al., 2025). Generative AI can offer immediate explanations, examples, summaries, writing assistance, and repeated responses without requiring students to disclose uncertainty to another person (Preiksaitis and Rose, 2023). These characteristics may make AI especially attractive to students who perceive peers, educators, or clinical supervisors as inaccessible, insufficiently supportive, or potentially judgmental (Fawaz et al., 2025; Klimova and Pikhart, 2025; Pham et al., 2025).

A systematic review of GenAI use in health professions education found that students most commonly used these tools for practice, inquiry, production, and knowledge acquisition (Pham et al., 2025). Discussion and collaboration were much less frequently represented, indicating that existing GenAI practices predominantly support individualized forms of learning (Pham et al., 2025). This pattern is relevant to the current findings taking note of that individualized AI interaction might provide assistance without (always) necesssitating interpersonal participation (Pham et al., 2025). For students experiencing academic and clinical learning loneliness, AI may therefore function as an alternative academic interlocutor rather than merely as an additional information source (Pham et al., 2025).

The relationship between learning loneliness and AI use should “after everything” not be interpreted as evidence that AI use is inherently harmful (Preiksaitis and Rose, 2023). Generative AI can support personalized explanations, autonomous practice, rapid feedback, and access to educational resources when it is used critically and under appropriate guidance (Preiksaitis and Rose, 2023; Fawaz et al., 2025; Pham et al., 2025). AI-supported environments may also strengthen self-regulated learning by assisting students during planning, performance, and reflection (Lan and Zhou, 2025). With that being said, these benefits may diminish when AI replaces rather than supplements human interaction, independent reasoning, and collaborative learning (Lan and Zhou, 2025; Pham et al., 2025). The current findings therefore concern the psychological and relational conditions associated with the frequency of AI use, not the educational value of every individual use. Borrowing cautiously from the ethical vocabulary of the health professions, a *primum non nocere* orientation can bear relevance to AI-supported education; technological assistance should not inadvertently “erode” learner agency, human connection and independent judgement which professional education seeks to cultivate.

The direct influence of academic wellbeing, academic incompetence, impostor syndrome, and overall academic self-perceptions on AI use remained significant after both mediators were included. These findings demonstrate partial rather than complete mediation and indicate that learned helplessness and learning loneliness do not fully explain the relationship between academic self-perceptions and AI use. Additional mechanisms may include perceived usefulness, ease of use, AI literacy, performance expectations, reassurance seeking, academic anxiety, and willingness to outsource demanding cognitive processes (Lan and Zhou, 2025). University students' attitudes and use of ChatGPT have been associated with perceived usefulness, ease of use, anticipated risks, and perceived psychosocial consequences.

The AI Use Scale employed in this study assessed the frequency and intensity of academic uses such as generating ideas, summarizing information, revising written work, solving course-related problems, and preparing for examinations (Yilmaz, 2026). The instrument did not classify each use as strategic, compensatory, dependent, academically inappropriate, or educationally beneficial (Yilmaz, 2026). This distinction is important mindful of the fact that AI dependency represents a conceptually more specific form of psychological and behavioral reliance that should not be inferred solely from the frequency of AI use (Li et al., 2025; Mazhar et al., 2026a). Thusly, the negative association between positive academic self-perceptions and AI use should not be interpreted as showing that lower AI use is necessarily better. Frequent AI use may represent productive support for one student but cognitive outsourcing, reassurance seeking, or avoidance of interpersonal assistance for another student (Preiksaitis and Rose, 2023; Lan and Zhou, 2025). The function and quality of AI engagement are thence as pivotal as its frequency (Pham et al., 2025; Yilmaz, 2026).

The specific indirect effects showed that learned helplessness independently mediated the relationships between all four academic self-perception variables and AI use. Academic wellbeing and positive overall academic self-perceptions were associated with lower AI use through reduced learned helplessness, whereas academic incompetence and impostor syndrome were associated with greater AI use through increased helplessness. This pathway suggests that students' academic self-evaluations may influence AI use partly by altering their belief that their own actions can produce successful outcomes. When students doubt their ability to influence outcomes, transferring difficult processes to AI may provide an immediate method of reducing uncertainty or avoiding anticipated failure (Zhai et al., 2024; Zhang and Xu, 2024; Lan and Zhou, 2025; Liu et al., 2026). Conversely, stronger perceptions of agency may allow students to use AI selectively without treating it as a substitute for their own academic capabilities (Zhang and Xu, 2024; Lan and Zhou, 2025).

Academic and clinical learning loneliness also independently mediated each relationship between academic self-perceptions and AI use. This finding demonstrates that the relational conditions of learning constitute a distinct mechanism beyond perceived control. This pattern highlights the relevance of belonging, accessible supervision, peer collaboration, and psychologically safe help-seeking in understanding AI use among health professions students (Ray et al., 2019; Chye et al., 2024; Chua et al., 2025; Mangali et al., 2025; Pham et al., 2025). Students who expect respectful and supportive responses to uncertainty may be more willing to consult human learning resources, whereas those anticipating judgement may prefer the privacy and immediacy of AI interaction (Chua et al., 2025).

An invaluable contribution of the present study is the integration of academic self-evaluation, perceived controllability, relational learning experiences, and AI use within a single explanatory framework. Academic wellbeing and positive overall academic self-perceptions were associated with lower learned helplessness, which was statistically associated with lower academic and clinical learning loneliness and, in turn, lower AI use. Academic incompetence and impostor syndrome showed the corresponding positive pattern across the same hypothesized statistical pathway, with greater helplessness, greater learning loneliness, and more frequent AI use. This sequence connects academic self-evaluation, perceived controllability, relational participation, and technology-related behavior within an integrated explanatory model.

Existing literature has generally examined academic self-beliefs, impostor experiences, learning support, self-regulation, and AI adoption as separate research areas (Chua et al., 2025; Lan and Zhou, 2025; Pham et al., 2025). The present study brings these domains together by proposing that negative self-evaluation may first weaken perceived control and subsequently arouse withdrawal from academic and clinical learning relationships. Relational disconnection may then increase the attraction of AI as an accessible, private, and non-judgmental source of assistance (Preiksaitis and Rose, 2023; Pham et al., 2025). Even supposing the cross-sectional design cannot portray that these processes necessarily occur in this temporal order, the consistency of the serial indirect association across all four predictor models indicates that the hypothesized ordering is compatible with the observed data. Either way, reverse or reciprocal relationships between learned helplessness and academic and clinical learning loneliness remain plausible. The results have implications for the integration of AI into health professions education (Pham et al., 2025). Institutional responses should not be limited to restricting AI tools or training students in technical prompting skills (Preiksaitis and Rose, 2023). AI literacy should also include critical evaluation of generated information, identification of inaccurate or biased content, academic integrity, privacy, attribution, and recognition of over-reliance (Preiksaitis and Rose, 2023; Zhai et al., 2024; Klimova and Pikhart, 2025; Pham et al., 2025). These competencies are specifically paramount in health professions education in the context of inaccurate or uncritically accepted information may eventually affect clinical reasoning, professional judgement, and patient safety (Preiksaitis and Rose, 2023; Zhai et al., 2024; Pham et al., 2025).

Responsible AI integration may also benefit from attention to the academic and relational conditions associated with students' technology use. Structured mentoring, accessible academic consultation, formative feedback, peer-assisted learning, and supportive clinical supervision represent plausible educational approaches for strengthening students' access to human learning resources yet their effects on the reasons for or frequency of AI use were not tested in the present study and therefore require direct evaluation in future research (Wynne et al., 2024; Pham et al., 2025). Educational environments may also seek to normalize uncertainty and communicate that asking questions constitutes a legitimate element of professional learning rather than evidence of incompetence (Rosenthal et al., 2021; Shinawatra et al., 2023; Chua et al., 2025). Supporting competence, agency, and belonging may therefore be considered a potentially relevant component of responsible AI education, all things considered, prospective research should intend to determine whether such approaches impact the “how, why, or how” of related preferences e.g., frequency of health professions students' uses of AI (Chye et al., 2024; Klimova and Pikhart, 2025; Lan and Zhou, 2025; Pham et al., 2025).

In light of all these itg would be fair to state that academic self-perceptions were associated with AI use amongst health professions students both directly and indirectly through learned helplessness and academic and clinical learning loneliness. Academic wellbeing and positive overall academic self-perceptions appeared to function as protective academic resources, whereas academic incompetence and impostor syndrome were associated with reduced control, relational disconnection, and more frequent AI use. Learned helplessness represented one statistical pathway linking negative academic self-evaluations with reduced perceived agency, while academic and clinical learning loneliness represented a complementary relational pathway linking reduced connection with human learning resources to more frequent AI use. The significant serial indirect effects were consistent with the proposed integration of students' academic beliefs, motivational experiences, learning relationships, and AI use, without establishing a definitive temporal or causal sequence among these constructs. Responsible AI integration in health professions education should consequently preserve student agency, reinforce supportive human interaction, and ensure that AI supplements rather than replaces reflective, collaborative, and clinically grounded learning (Preiksaitis and Rose, 2023; Zhai et al., 2024; Klimova and Pikhart, 2025; Lan and Zhou, 2025; Pham et al., 2025).

## 5 Limitations and future research

This study employed a cross-sectional research design; therefore, the relationships among academic self-perceptions, learned helplessness, academic and clinical learning loneliness, and AI use should not be interpreted as causal (Spector, 2019). More specifically, the serial mediation analysis cannot establish temporal precedence between learned helplessness and academic and clinical learning loneliness. Whereas the ordering tested in the present model was theoretically specified, reverse and reciprocal relationships are also plausible. Future longitudinal or cross-lagged studies should therefore examine the temporal and potentially reciprocal relationships between these constructs. A further limitation concerns the reliance on self-report measures administered within the same questionnaire and at the same time point, which may have introduced common method bias. Harman's single-factor test did not indicate a dominant single factor, *au contraire*, this procedure cannot exclude common method effects (Podsakoff et al., 2003). Future studies might wish to address this limitation through incorporating multiple data sources, temporally separated measurements, or more robust statistical approaches for evaluating method-related variance. In consideration of the fact that the three dimensions of academic self-perceptions were scrutinized in distinct mediation models, the findings obtained do not establish the unique contribution of each dimension after controlling for the other dimensions.

The usage of convenience sampling and the inclusion of students from a single health sciences university also require caution when extending the findings to other institutions and health education programmes. In addition to this, academic and clinical learning loneliness was assessed using a newly developed measure. Even though the scale demonstrated satisfactory psychometric properties in an independent development sample, further validation across institutions, disciplines, and cultural contexts is warranted. Another indispensable sort of limitation concerns the interpretation of AI use. The AIUS assesses the frequency and intensity of academic AI use rather than the specific function, motivation, or meaning of individual AI-use episodes. This distinction is crucial making allowance for that AI dependency involves a more psychologically and behaviorally meaningful form of reliance, including the delegation of judgment or functions to AI, and therefore cannot be inferred from usage frequency alone (Mazhar et al., 2026b). Consequently, frequent AI use cannot be interpreted as inherently compensatory, avoidant, dependent, or problematic; similar levels of use may reflect productive learning support for one student and reassurance seeking, cognitive outsourcing, or avoidance of interpersonal help-seeking for another.

When all said and done, the study offers an integrated model for research-to-come by examining AI use among health professions students together with academic and relational variables. Future studies should test this model with students from different universities and a wider range of health programmes. Longitudinal research can carry the potential to further clarify the direction of the relationships over time (Spector, 2019; Lan and Zhou, 2025). In addition, distinguishing among supportive, compensatory, and intensive forms of AI use can help with a more detailed understanding of the academic and psychological conditions under which students turn to AI (Zhai et al., 2024; Zhang and Xu, 2024; Pham et al., 2025; Yilmaz, 2026).

## Data Availability

The data analyzed in this study is subject to the following licenses/restrictions: The original data supporting the findings of this study will be made available by the author upon reasonable request, without undue restriction. Requests to access these datasets should be directed to Pinar Ayyildiz, pinarayyildiz@yahoo.com.
